# Comparing the experiences of cancer survivors living with sleep disturbances between differing levels of psychological distress: a qualitative study

**DOI:** 10.1186/s12888-024-06344-4

**Published:** 2024-12-02

**Authors:** Julia Chan, Danielle Wing Lam Ng, Richard Fielding, Wendy Wing Tak Lam

**Affiliations:** 1 School of Public Health, Centre for Psycho-Oncology Research and Training, University of Hong Kong, Hong Kong, China; 2 Li Ka Shing Faculty of Medicine, Jockey Club Institute of Cancer Care, University of Hong Kong, Hong Kong, China

**Keywords:** Sleep disturbance, Psychological distress, Qualitative, Experiences, Psycho-oncology

## Abstract

**Background:**

Psychological distress often co-occurs with sleep disturbances; but the specific mechanisms linking the two remain unclear. A qualitative study explored perceptions and factors associated with sleep disturbances in cancer survivors between patients with varying levels of psychological distress.

**Methods:**

Thirty-three Cantonese speaking mixed type cancer survivors were recruited from a community cancer care program. Participants that scored > 5 on the Pittsburgh Sleep Quality Index and had non-clinical or borderline to clinical levels of psychological distress underwent semi-structured interviews. Interviews were analyzed using grounded theory.

**Results:**

Common triggers of sleep disturbances included unresolved treatment side-effects, intrusive thoughts about cancer and fear of cancer recurrence or progression, poor sleep hygiene and a lack of routine. Those with higher levels of distress exhibited more worry about recovery after cancer. Further, they engaged in thought suppression and experienced meta-worry about negative emotions and worry of poor sleep impacting recovery and cancer progression. They commonly exhibited high sleep reactivity and were observed to have limited social support. In contrast, those with low distress adopted better adaptive mechanisms, including a changed commitment to prioritizing health and positive reappraisal of their recovery progress. Self-distraction was used to cope with sleep disturbances and they had fewer expectations of good sleep quality.

**Conclusions:**

Findings provided insights into the suitability of interventions for patients with sleep disturbances. Interventions targeting maladaptive emotion-focused coping may be more effective in addressing sleep disturbances in cancer survivors with higher distress. Interventions adopting a stepped-care approach may be advantageous in managing sleep disturbances by catering for varying levels of distress.

**Supplementary Information:**

The online version contains supplementary material available at 10.1186/s12888-024-06344-4.

## Introduction

Sleep disturbance is a highly prevalent symptom experienced by cancer patients; pooled prevalence reports have indicated that 60% experience sleep disturbance during active treatment and 59.7% more than 3 months post-treatment [1]. Furthermore, high levels of sleep disturbance were observed to persist in 30% of cancer survivors for two years after treatment completion [2]. The negative impact of sleep disturbance extends beyond quality of life [3]; long-term sleep disturbance has been linked with poorer health consequences, including increased morbidity and mortality generally [4], as well as adverse effects on tumor growth and cancer progression in cancer patients specifically [5].

Psychological distress is another prevalent condition affecting cancer patients, and has been linked to sleep disturbance [6]. Psychological distress has been reported in one in two cancer patients (52%) across multiple cancer groups [7]. The highest levels of psychological distress are typically observed during cancer diagnosis and treatment periods [8, 9], and while psychological distress shows a decreasing trend after cancer diagnosis [10], 12–19% of women report experiencing no declines in chronic distress after completing breast cancer treatment [11–13].

Significant overlap of sleep disturbance and psychological distress is reported in cancer survivors. Depressive symptoms have been identified as a risk factor for sleep disturbance in a systematic review and meta-analysis examining breast cancer survivors [14]; and in mixed type cancer patients at two years post-treatment [15] and at 9 years post-diagnosis [16]. Likewise, anxiety was associated with sleep disturbance in long-term prostate cancer survivors [17]. Longitudinal studies have further highlighted the lasting impact of psychological distress on sleep disturbance across various cancer types, underscoring the complex relationship between the two. Initial high levels of depressive and anxiety symptoms measured within 4 months of a new diagnosis of breast cancer were associated with high levels of chronic sleep disturbance up to one year post-diagnosis [18]. Greater increases in depression predicted subsequent increases in sleep disturbances during the first year of diagnosis in ovarian cancer patients [19], and baseline depressive symptoms predicted persistent sleep disturbances during two years post-treatment [2]. Conversely, lagged effects of poor sleep increased both fatigue and subsequently depressive symptom levels in ovarian cancer patient during treatment, suggesting the relationship to be a two-way influence [6]. Worsening anxiety among colorectal cancer patients was found to be associated with increasing sleep disturbance up to 10 months following diagnosis [20]; and baseline trait anxiety measured before treatment predicted lower sleep quality in breast cancer survivors at 2 year follow-up [21].

However, there are instances where sleep disturbance experienced by cancer survivors prevails without anxiety or depression. For example, depression and anxiety symptoms are associated with sleep disturbance near diagnosis and treatment [22–24], but the effect of anxiety on sleep disturbance was not found to sustain longitudinally after treatment [2, 19]. Another longitudinal study found anxiety, but not depression, predicted sleep disturbances in breast cancer survivors at 2 years post-chemotherapy [25]. As a result, the relationship between sleep disturbance and psychological distress cannot be assumed to be mutually reinforcing in cancer survivors.

While several frameworks and models have attempted to conceptualize the psychological mechanisms involved in the development and maintenance of sleep disturbance in general populations, variations in mechanistic properties between proposed models leave the exact nature of the relationship between sleep disturbance and psychological distress, particularly in the context of cancer survivors, unclear. For instance, Spielman et al. [26] proposed that the presence of anxiety and depression disorders may serve as a precipitating factor that triggers the onset of insomnia, while Espie [27] and Harvey [28] suggested that a combination of selective attention towards worries about sleep and dysfunctional sleep-related beliefs, such as negative consequences of poor sleep, leads to heightened arousal and psychological distress, which ultimately results in sleep interference.

Given that the relationship between sleep disturbance and psychological distress is unclear, exploration of the relationship between the two is needed to build a conceptual framework for the development and maintenance of sleep disturbance in a cancer setting. Sleep disturbance is likely to be affected by others factors such as employment status [2, 29], lasting treatment related side-effects including pain and hot flashes [14, 19, 30], and disruptive chronobiological effects on circadian rhythms [31] as a result of chemotherapy. However, given that different factors may impact sleep quality at different times across the trajectory of cancer survivorship, it is important to understand the context to which such factors may trigger or perpetuate sleep disturbances in order to understand the mechanisms behind sleep disturbance in the presence or absence of psychological distress. For example, disruptions of circadian rhythms resulting from chemotherapy are likely to be less impactful towards the end of treatment [32] and psychological distress is likely to play a larger role in perpetuating chronic sleep disturbances after treatment completion [2]. Identifying the differences in the experiences of sleep disturbances in cancer survivors between different distress levels through qualitative analysis can help to clarify these mechanisms. Existing qualitative studies have predominantly focused on the contributing factors and negative consequences of sleep disturbance [33–38] without exploring the potential underlying processes of sleep disturbance, such as the perception of sleep, which has only been covered by a handful of qualitative studies [39–42].

We report a qualitative study implemented to provide insight into the differences in experiences of sleep disturbance between cancer patients with varying levels of psychological distress in order to illuminate factors involved in developing and maintaining sleep disturbance.

## Methods

### Design

Given that psychological distress and perceptions of sleep are known to contribute to sleep disturbance, a qualitative study was conducted to investigate the variations in patient responses across different levels of distress with the aim of gaining a better understanding of the underlying processes leading to the development of sleep disturbance. Using a descriptive-interpretive approach, we compared the lived experiences of cancer survivors with sleep disturbance between those reporting non-clinical levels of psychological distress and those reporting borderline to clinical levels of psychological distress.

### Participants

Purposive sampling was used to select cancer survivors who had attended a community-based multidisciplinary cancer care program between September 2022 and August 2023. Ethics approval was granted by the Institutional Review Board of the University of Hong Kong/Hospital Authority Hong Kong West Cluster (No: UW 22–595). Eligible patients were Cantonese and Mandarin-speaking Chinese patients aged 18 years or older, diagnosed with cancer without metastatic disease. Given that sleep disturbance was observed to persist in 30% of cancer survivors for two years after treatment completion [2], the qualitative study also aimed to capture experiences of sleep disturbances for patients that had completed curative primary and adjuvant treatments within two years, during the early phase following treatment completion. Patients were assessed for sleep disturbances and psychological distress using the Pittsburgh Sleep Quality Index (PSQI) [43] and the Hospital Anxiety and Depression Scale (HADS) [44]. Patients that either have 1) poor sleep (PSQI > 5) and non-clinical levels of anxiety or depression (HADS ≤ 7) 2) or poor sleep (PSQI > 5) and borderline or clinical levels of anxiety or depression (HADS ≥ 8) were identified as eligible participants. Patients that were regularly seeing psychiatric services, or attended workshops or interventions targeting sleep or psychological distress were excluded. Patients with linguistic or intellectual difficulties or receiving active adjuvant treatment (excepting hormonal treatment as it is often used as adjuvant therapy to reduce the risk of cancer recurrence and is typically taken for at least 5 years) were excluded.

### Procedure

Patients were approached after attending the community cancer care program and were asked if they would like to take part in an interview on their experiences of sleep disturbance. Willing participants completed a screening interview and were recruited if they met eligibility criteria. Written consent was obtained, and each participant was scheduled to complete an in-depth, semi-structured, recorded interview of approximately 45 min in duration that was either conducted via a phone call or Zoom, a digital video conferencing platform. The interviewer (J.C.) was a doctoral candidate with a specialization in psycho-oncology and behavioral health. The semi-structured interview guide included questions regarding the sleep disturbance experience before and after cancer diagnosis, factors perceived to impact sleep quality, strategies that were used to manage sleep disturbances, thoughts and feelings that arise when poor sleep occurs, beliefs, attitudes and expectations towards sleep, perceived need to seek professional help in managing sleep disturbance and understanding of sleep hygiene (Appendix A). To minimize the burden on the participants, participants were not asked to review the transcripts. To ensure that the meanings attributed to the participants' responses accurately reflected their intended message, the interviewer repeatedly reconfirmed their understanding and interpretation of the responses throughout the interviews. This involved summarizing key points and confirming with participants to ensure their responses were correctly understood in real time. The sample size was determined by data saturation within each of both groups, where additional data did not lead to any new emergent themes [45].

### Data analysis

All digitally recorded interviews were transcribed verbatim in Cantonese/traditional Chinese. Transcripts were then analyzed and coded using the grounded theory technique [46]. Data was subjected to constant comparative analyses. The analytical process was based on immersion in the data and repeated sorting, coding, and comparison. Each interview was examined line-by-line and subsequently sorted into code components by two coders (J.C. & Y.S.). Each code component from both coders was compared to each other to ensure that the codes were mutually exclusive and to identify any possible connections, which were then clustered to form broader categories or themes. The NVIVO software, designed for organizing and processing qualitative data, was used to facilitate data coding and analysis. The properties of the studied concepts were then hierarchically derived [47]. Following systematic organizations of code components and themes, the data were cross-checked by senior team members (W.W.T.L. & D.W.L.N.) experienced in qualitative research to increase the validity of theoretical constructs through repeated discussions. All analyses were performed in the original language of Cantonese.

## Results

### Participants

Thirty-three cancer patients consisting of breast, gynecological, head and neck, colorectal and lymphoma cancer participated in the study. On average, the patients were diagnosed 15 months prior to the time of recruitment. All patients presented with clinical levels of sleep disturbances (PSQI > 5), of whom 13 reported borderline or clinical levels of psychological distress (HADS ≥ 8), and 20 reported non-clinical levels of psychological distress (HADS ≤ 7). Patients in the non-clinical and borderline to clinical distress group reported a global PSQI score of 9.75 ± 3.36 and 11 ± 3.34 respectively. The non-clinical distress group reported a mean anxiety and depression score of 2.00 ± 1.72 and 1.75 ± 1.07; while the borderline clinical group reported 9.23 ± 1.54 and 7.38 ± 4.19 respectively. Additional demographic and clinical data are presented in Tables 1 and 2 in case-by-case and quantitative formats respectively. Pertinent narrative data are presented in Tables 3, 4, 5, referenced in text below by italicized numbers, (e.g. Table 3, 1.1).

**Table 1:** Personal and clinical characteristics of the participants (case-by-case)

Case	Age	Gender	Marital status	Education	Employment	Cancer type	Recurrence	Type of treatment received	Psychological distress level
1	37	Female	Married	Secondary	Housewife	Gynecological	No	Surgery, chemotherapy	Borderline-Clinical
2	71	Female	Married	Secondary	Part-Time	Breast	No	Surgery, hormonal therapy	Non-Clinical
3	57	Female	Single	Tertiary	Retired	Breast	No	Surgery, hormonal therapy	Non-Clinical
4	52	Female	Married	Secondary	Full-Time	Gynecological	No	Surgery, chemotherapy	Non-Clinical
5	42	Female	Married	Secondary	Unemployed	Breast	No	Surgery, chemoradiotherapy, hormonal therapy	Non-Clinical
6	60	Male	Married	Secondary	Full-Time	Head and Neck	No	Radiotherapy	Non-Clinical
7	66	Female	Married	Secondary	Retired	Lymphoma	No	Radiotherapy, target therapy	Borderline-Clinical
8	58	Female	Married	Primary	Housewife	Head and Neck	No	Chemoradiotherapy	Borderline-Clinical
9	36	Male	Married	Tertiary	Full-Time	Head and Neck	No	Chemoradiotherapy	Non-Clinical
10	67	Female	Single	Secondary	Retired	Head and Neck	No	Surgery, radiotherapy	Non-Clinical
11	68	Female	Married	Secondary	Housewife	Head and Neck	No	Surgery, chemoradiotherapy	Non-Clinical
12	69	Female	Married	Secondary	Housewife	Head and Neck	No	Surgery, radiotherapy	Non-Clinical
13	64	Male	Married	Tertiary	Retired	Head and Neck	No	Chemoradiotherapy	Non-Clinical
14	66	Male	Married	Secondary	Unemployed	Head and Neck	No	Surgery, chemoradiotherapy	Non-Clinical
15	51	Male	Married	Secondary	Full-Time	Head and Neck	No	Chemoradiotherapy	Borderline-Clinical
16	54	Male	Married	Secondary	Full-Time	Head and Neck	No	Chemoradiotherapy	Non-Clinical
17	73	Female	Married	Primary	Housewife	Head and Neck	No	Surgery, radiotherapy	Non-Clinical
18	65	Male	Married	Secondary	Retired	Head and Neck	No	Surgery, radiotherapy	Non-Clinical
19	75	Female	Married	Secondary	Retired	Breast	No	Surgery, chemoradiotherapy	Non-Clinical
20	50	Female	Married	Tertiary	Unemployed	Breast	No	Surgery, radiotherapy, hormonal therapy	Non-Clinical
21	59	Female	Married	Secondary	Housewife	Breast	No	Surgery, hormonal therapy	Borderline-Clinical
22	59	Female	Married	Tertiary	Retired	Breast	No	Surgery, chemotherapy, target therapy, hormonal therapy	Non-Clinical
23	52	Female	Married	Secondary	Unemployed	Breast	No	Surgery, chemotherapy, target therapy, hormonal therapy	Borderline-Clinical
24	35	Female	Married	Tertiary	Unemployed	Breast	Yes	Surgery, radiotherapy, hormonal therapy	Borderline-Clinical
25	63	Female	Married	Secondary	Retired	Breast	No	Surgery, chemotherapy, target therapy	Borderline-Clinical
26	48	Female	Married	Tertiary	Full-Time	Breast	No	Surgery, chemoradiotherapy, hormonal therapy	Borderline-Clinical
27	69	Female	Married	Primary	Housewife	Breast	No	Surgery, radiotherapy, hormonal therapy	Borderline-Clinical
28	53	Female	Married	Secondary	Full-Time	Gynecological	No	Surgery	Non-Clinical
29	59	Male	Married	Tertiary	Full-Time	Colorectal	No	Surgery, chemotherapy	Non-Clinical
30	56	Female	Married	Secondary	Unemployed	Gynecological	No	Surgery, chemoradiotherapy	Non-Clinical
31	54	Female	Single	Secondary	Full-Time	Gynecological	No	Surgery, chemotherapy	Borderline-Clinical
32	53	Female	Married	Secondary	Part-Time	Gynecological	No	Surgery	Borderline-Clinical
33	50	Female	Single	Secondary	Part-Time	Gynecological	No	Surgery	Borderline-Clinical

**Table 2:** Personal and clinical characteristics of the participants (quantitative)

**Demographic data**	Participants (*n* = 33)
Gender	
Male	8 (25.0%)
Female	25 (75%)
Age	
20–39	3 (9.1%)
40–59	17 (51.5%)
60–79	13 (39.4%)
Marital status	
Married	29 (87.9%)
Single	4 (12.1%)
Education level	
No primary/Primary	3 (9.1%)
Secondary/tertiary	30 (90.9%)
Employment	
Full-time/part-time	12 (36.4%)
Housewife	7 (21.2%)
Retired/unemployed	14 (42.4%)
Distress level	
Non-clinical	20 (60.6%)
Borderline to clinical	13 (39.4%)
**Clinical data**	
Cancer Type	
Breast	12 (36.4%)
Head & Neck	12 (36.4%)
Gynaecological	7 (21.2%)
Colorectal	1 (3.0%)
Lymphoma	1 (3.0%)
Treatment received	
Surgery	26 (78.8%)
Chemoradiotherapy	11 (33.3%)
Chemotherapy	7 (21.2%)
Hormonal therapy	10 (30.3%)
Radiotherapy	9 (27.3%)
Target therapy	4 (12.1%)

### Nature of sleep disturbance (Table 3, 1.1–1.3)

Regardless of the psychological distress level, all patients reported similar patterns of sleep disturbance, including nocturnal waking, long sleep onset latency, and shortened sleep duration.

Patients frequently reported greater sleep onset latency compared to before their cancer diagnosis, sometimes waiting 30 min to 4 h after getting in bed before sleep onset (*1.1*). Frequent nocturnal wakening with difficulty in resuming sleep after waking in the middle of the night (*1.2*), together with long sleep onset latency effectively reduced the nighttime sleep duration (*1.3)* were common problems. Some patients reported large reductions in sleep duration after cancer diagnosis compared to before, with a reduction from approximately eight to five hours (*1.3)*.

**Table 3:** Nature of sleep disturbance and common triggers in cancer survivors, narrative quotes

1. Nature of sleep disturbance	
1. Long sleep onset latency	Case 2: It takes a long time (to go to sleep). Sometimes it may take around half an hour or even an hour and a half. I can get very tired, and sometimes I really feel very sleepy, but when I go to bed, I just can't fall asleep... Case 7: I usually go to bed and sleep around 11:30 pm. But sometimes I'll toss and turn until a certain hour... and sometimes I can't fall asleep until even 2:00 am.
2. Nighttime awakenings	Case 10: After falling asleep, I usually sleep for about one to two hours. I wake up automatically. It’s like that every day... after waking up, sometimes it's very easy to fall asleep again. Sometimes it's hard to fall asleep again. Like these two days, I woke up at two o'clock and had to wait until four or five o'clock to fall asleep. After falling asleep again, I can sleep for about three hours! Then I wake up around seven or eight o'clock! Case 15: On average, I used to sleep until dawn (before cancer) ... But now, I can't do that anymore. Maybe I wake up around 2 o'clock and can't go back to sleep.
3. Shortened sleep duration	Case 6: At the start, I sleep well for the first few hours. Even if it is only a few hours, I sleep quite deeply. But then, it doesn’t last very long, I wake up after a while. (I have) five or six hours of sleep, it’s very short, because before my treatment, I used to sleep for eight or nine hours every day. It's a big difference.
2. Common triggers of sleep disturbance	
1. Treatment-related side effects	Case 20: Because I have been taking hormonal medication, and now I have to take it for five years. These medications, I feel, have some slight reactions on the body, like sweating... So even with the air conditioning on, I still sweat and wake up. Case 1: I'm not sure why this is happening. This situation has been occurring frequently at night for several hours, and I didn’t even experience it during the period of chemotherapy... I have already completed chemotherapy for one or two months. I don't know why my feet and hands are still numb. I have been experiencing these symptoms every now and then for the past few days. Case 7: It's really hard to sleep continuously, and I wake up every two hours, that's how it is. It's been like that all the way (since treatment completion). After getting the injection, I feel tired...When you're tired, (I thought) you can sleep longer, but you still wake up every two hours... At night, I feel even more tired, but I still can't fall asleep in bed. Case 10: Life in the hospital has a significant impact on my sleep. Even at midnight or at 11-12 pm, the hospital is still operating normally. Some people walk around and make a lot of noise, and the lights are always turned on. This disrupts the normal sleeping pattern and makes it abnormal! It makes it difficult to fall asleep, and people end up sleeping around two or three in the morning. ... I lived in the hospital for two months... I suspect (the reason that I still sleep poorly) that during these two months, I've gotten used to this kind of situation! It's become a habit. For example, you can sleep during the day if things get messed up... but I can't even sleep during the day!
2. Intrusive thoughts about cancer and fear of cancer recurrence	Case 1: Because I had a major surgery where they had to cut me open. I had to undergo it twice! So, I would suddenly wake up in the middle of the night. Since chemotherapy, these nightmares have reduced! But I still wake up frequently! At least for several months, continuously, I would be scared awake. Chemotherapy is tough, with even more side effects. And dealing with different reactions and effects of each chemotherapy session... It's scary! Actually, the surgery was also incredibly painful. (laughs) Because being cut open is like being butchered like a pig. The incisions are so large! It's terrifying. It feels like being stapled with large staples, terrifying to death. Case 27: I've completed treatment, so it's okay! But every now and then, I think back, like when I can't sleep, I suddenly remember the radiotherapy! The preparation before surgery! And then, it repeats again! ...But it doesn't happen every day, actually. I try not to think about these things! Because I feel like it's already in the past! But sometimes when I can't sleep, they come back, they resurface... Case 5: After getting diagnosed with cancer, I kept having nightmares. It started during chemotherapy. Those nightmares were like, it felt like my grandparents were calling me back, but they're not here anymore. And other people were calling me too, so my heart was filled with fear... It happens once every two or three weeks. Plus, I was no longer considered in the early stage as I was already stage 3 when I was diagnosed, so I became scared. So, I thought, ‘Are they calling me to leave... Case 16: For example, when I had to go back for a check-up, it was very difficult to sleep in the week before. There are things to worry about, especially about my own illness, so I feel particularly nervous before getting the results. If the results come out fine, then I can sleep well. I'm also nervous, overly nervous. I'm really afraid of recurrence. Everyone talks about recurrence as if it's instantly bye-bye (death). So, I feel extremely anxious. Case 20: I might think about, or worry about, will it (cancer) recur? Or maybe I will think about…will I burden my family? Or maybe I’ll think about…what about returning to work after recovery, will my body be able to handle that? Will I be able to adjust? Can I keep continuing on? So, there are many things to think about.
3. Poor sleep hygiene	Case 28: Actually, half of the reasons why (I am unable to sleep) is because of my husband's snoring! You get really frustrated! Sometimes, when he snores, he wakes me up! ... I really can't fall asleep, I have tried to, but what can I do? He has already gone to bed a lot later than me, (coming into bed) a lot later! But he still wakes me up, I can't avoid it. Case 23: After finishing work, I have to take a look (at my phone), because sometimes I work at night, and it becomes very late by the time I finish. I can't fall asleep immediately, so I take my phone out and take a look. I used to have this habit, thinking it's a way to reduce stress! But actually, I know this will affect my sleep. So, I try not to do it.
4. Lack of structured daily routine	Case 17: …Well, it started when I retired, as I had more time, and I couldn't sleep well. But if I did some chores, did some things, did more housework, then I could sleep better. If there was nothing to do, I’m done for. I would sleep until four or five in the morning and wake up. Before retirement? When I was working, I could sleep as soon as I laid down. Case 13: After retirement, I don't feel particularly tired during the day, unless you do something that makes you tired... After a day's work I will get tired, so I will fall asleep faster.

### Common triggers of sleep disturbances (Table 3, 2.1–2.4)

Common triggers of sleep disturbance were identified for both patients with non-clinical or borderline to clinical levels of distress. These included treatment-related side effects, intrusive thoughts of cancer, fears of cancer recurrence, and non-cancer-related factors.

#### Treatment-related side effects (2.1)

Treatment brought upon a myriad of physical side effects which contributed to sleep disturbances. For example, a breast cancer patient (Case 20) spoke about how treatment-induced menopausal symptoms, hot flushes and sweating, lead to frequent nocturnal wakening. Persistent symptoms of peripheral neuropathy, including numbing and prickling at night, were experienced by a gynecological cancer patient who had completed chemotherapy (Case 1). Apart from the physical side-effects of treatment contributing to sleep disturbance, patients also perceived treatment to have disrupted their sleep–wake cycle, which was most commonly reported by those who had undergone chemotherapy or radiotherapy. For example, a patient with lymphoma (Case 7) experienced a significant deterioration of sleep quality after receiving a combination of chemotherapy and targeted therapy, which persisted beyond treatment completion.

Extended periods of hospitalization as a result of treatment complications negatively impacted sleep patterns for one patient. Even after being discharged, a patient with head and neck cancer (Case 10) faced difficulties in falling asleep at night, and even struggled to fall asleep in the day when attempting to make up for lost sleep.

#### Intrusive thoughts about cancer and fear of cancer recurrence (2.2)

Irrespective of levels of distress, intrusive thoughts about the cancer treatment process, or fears of cancer recurrence and progression were common. Patients reported nightmares disrupting sleep quality in the form of vivid flashbacks regarding the cancer treatment process (Case 1) which they perceived as traumatic, painful and frightening. Recalling the trauma of treatment also occurred at night when distractions are fewer, causing difficulty in initiating sleep (Case 27). One patient (Case 5) reported having recurring nightmares of deceased relatives during chemotherapy, which continued regularly after treatment completion. These nightmares often triggered thoughts of the severe consequences of cancer recurrence. Anticipatory anxiety affected sleeping as regular check-ups with oncologists approached, triggering fear of recurrence, adding to existing anxiety (Case 16). Fears of recurrence also accompanied other worries concerning the potential burden that recurrence may have on family members on top of adjustment concerns to cancer, such as the implications of their ability to return to work, which often kept their minds active before falling asleep (Case 20).

#### Poor sleep hygiene (2.3)

Non-cancer-related factors, such as poor sleep hygiene or disruptive sleeping environments had also contributed to poor sleep. For example, a partner's persistent snoring was a source of frustration for the patient as it has consistently prevented them from falling asleep (Case 28). Further, a considerable number of patients admitted to the habit of scrolling their phones in bed. Although the patient was aware of the detrimental effects on their sleep quality, they struggled to break free from the habit, as they used it as a way to manage stress after a busy day of work (Case 23).

#### Lack of routine (2.4)

Many patients noted a lack of employment as having a noticeable effect on sleep quality. For example, a patient (Case 17) observed that having no plans in the day would result in a shortened sleep duration to which they would wake up too early in the morning. Meanwhile, completing the chores and engaging in housework helped them to sleep better. Another perceived that the physical and mental demands of their jobs before retirement would naturally lead to increased daytime fatigue and ultimately result in better sleep quality at night (Case 13).

### Differences in experiences of sleep disturbances in patients between varying distress levels

**Fig. 1:**
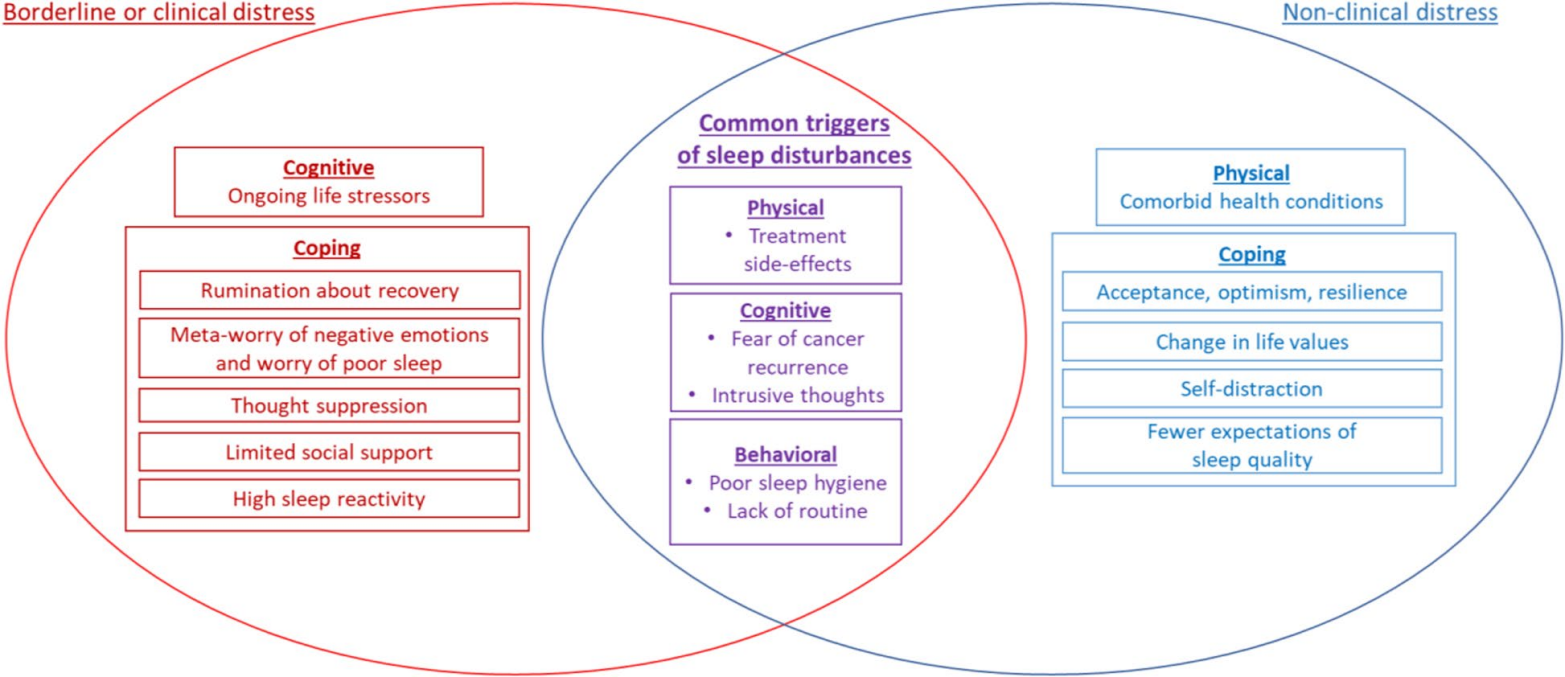
Triggers of sleep disturbances and coping responses of varying distress level

Table 4 documents sleep disturbance experiences in borderline to clinical distress (Table 4.A) versus non-clinical distress (Table 4.B). Differences and similarities in experiences between both groups are visualized in Fig. 1.

**Table 4:** Differences in experiences of sleep disturbances in patients between varying distress levels, narrative quotes

A. Borderline or clinical psychological distress			
1. Rumination and worries about recovery and returning to normal		Case 8: Half a year ago, I might have just cried because the experience is so isolating and painful...Even though I have completed treatment, I still have a lot of side effects that make me uncomfortable. These side effects will be with me for the rest of my life, as the doctor says. … You’ll feel very distressed, but it's like you can't do anything about it…If you take painkillers, maybe it will relieve the symptoms for a few hours or so, but you can't keep on taking it, right? Case 25: After chemotherapy, I feel like I have become weak, and I was already nervous, and can't relax, and my muscles feel so uncomfortable. There are numerous side effects! As a result, I feel stressed and have to take sleeping pills to sleep. I can't sleep at all. Every time I try to sleep, I wake up immediately!... During treatment, I wondered why my body hurts, it's like I can't accept the pain, it's such a painful pain, it hurts so much… I started to think, am I going to die? I became scared and couldn’t think straight, I would think of lot of things… I was scared, what if I can’t walk? What if I can’t recover? What if my condition never improves! With this worry. I'd rather die...I thought like that! Case 15: Especially after completing it (treatment)... I had to rely on medicine, like painkillers. It's (throat) been continuously ulcerated... it's been a few months, it's really painful... (laughs) At that time, I was thinking I don't know if I will recover well. Compared with other patients, we all have the same symptoms, everyone with nasopharyngeal cancer. But they don't have pain as intense as mine, they recover faster. Every time I see them, it’s when we bump into each other (to receive check-ups). She also says she has pain, but it's not as continuous and as long as mine.	
2. Worry about negative emotions affecting cancer progression and subsequent thought suppression		Case 8: When you think about it (side effects of cancer), you will worry, and when you worry, it will affect you. So, you have to find a way to let go of these emotions, not to be overly worried. ... Therefore, it's even more important to let yourself know that you must learn how to make yourself happier, because (negative) emotions, I've heard that they can affect your hormonal balance... You hear your own thoughts and you know that you’re thinking too much, many people have the same situation. So now you have to tell yourself that you really... hey, you're okay now, don't always think that something will happen tomorrow. Don't think like that, otherwise I'll always be unhappy, but I can't stop myself from thinking. Case 1: After getting angry, I think to myself, "Oh no! Did I make my condition worse (by getting angry)?" I constantly think about this question, whether it will affect my chemotherapy process, what if I can't receive it. I was really worried! So, in the end, I tried to calm myself down, and not think too much, and not argue with him (husband)! Sometimes, I tell my husband, "Can you help me out with some things? Otherwise, I might explode..." It's really scary for me. So, I try my best not to get angry, because getting angry is not good for my body.	
3. Worry about poor sleep affecting recovery and subsequent pressure to achieve good sleep		Case 7: Because I am a cancer patient. Even though I have completed my treatment, I know that sleep is very important for people like us who are recovering. So, I feel pressured about getting enough sleep. I worry that if I don't sleep well, it might increase the chances of recurrence. There are so many things to worry about, but I can't control them. Case 24: Since I’ve had recurrence... plus knowing my own background... it's obvious that I’m high-risk! So naturally, it means that you have to constantly pay more attention to this matter (sleep quality). Case 23: When I lie down to sleep, I will immediately remind myself... You can’t do this! You need to sleep! The doctor said I need more sleep! I never get enough sleep! What should I do? This needs to stop! Today, I'll try my best to take a nap during lunchtime. …I just keep thinking about it... If I don't get enough sleep, I will think "I can't sleep, I have to go to sleep!" The more I think about it, the worse it gets. I can't fall asleep... Case 1: The more scared I am (about not being able to sleep), the harder it is to fall asleep. The more worried I am about it, the more terrifying it becomes. The more I think about it, the harder it is to fall asleep. Because sometimes I think to myself, 'Oh no! I didn't sleep well last night, will it continue?' Sometimes it (unable to sleep) happens for several nights in a row!" Case 27: It’s really frustrating! I’m on my bed thinking, "Why can't I fall asleep? (laughter) I should be very comfortable already!” Case 24: If I am sleeping and I feel pain here, then naturally, I would think... Could there be something wrong?	
4. High sleep reactivity to ongoing life stressors and lack of social support		Case 26: Maybe like a week ago, suddenly the company gave me a task to do. I felt very anxious and worried, and then I had a nightmare at night! I dreamt of my boss, telling me to go to work. I think I get nervous easily. Case 27: Sometimes I can’t sleep, it’s because I'm afraid for my children! I’m afraid that something unexpected might happen, I'm really scared! ... That's why I try to think about it less. But I'm still worried. My daughter is a flight attendant! When she flies, I'm a bit worried! Case 32: I know that if I keep thinking about (plans for the next day) for a long time, and I know that it's thinking about it for too long, I won't be able to sleep all night, and I'm very afraid of not being able to sleep. Because if that happens, I won't be able to do anything the next day. Case 1: The hospital introduced a type of medication to me... so, I tried it... But because I didn't have the support of my husband, I got angry. I argued with my husband!...That medication is too expensive, it costs HK$45,000 (~US$5,770) per month. So, I felt that my husband didn't understand me as a patient. I really want the support of a family member!…At the very least, he should comfort me, right? Instead of constantly arguing with me about the issue of money... I argued with him, even during my chemotherapy! I couldn't sleep properly during that time, I was very angry! Then, after I got angry, I thought to myself, "Am I making my condition worse?" Case 31: When I can't sleep, I will think about a lot of things (laughs). It happens all the time. The biggest problem is my brother, because my brother has intellectual disabilities. We are both unmarried. But, my parents are old! And because of me, suddenly there was this problem (cancer)…Before I had cancer, he was already a stressor. Because my brother needs taking care of! I never thought that I would have this illness, and it’s given me more stress!	
B. Non-clinical psychological distress			
1. Acceptance, resilience and optimistic view of cancer and treatment experience		Case 11: And the chemotherapy staff were very good to me too. I'm very grateful, really. In the later stages, they would say, "Oh, you might experience some discomfort or damage to your neck due to the radiation therapy." Fortunately, I only had some slight burns and darkening on my neck, but no major damage, so that's lucky. Yes. And the care they provided was really good, and it changed my initial fear. Then I realized it wasn't as difficult as I had imagined. I have accepted it, from being afraid at the beginning to being okay. That's my mindset. Case 14: Because I have many health conditions myself, firstly, my upper respiratory tract is sensitive, I easily cough and have a runny nose, and then later on, I was diagnosed with cancer and then rheumatism. I haven't worked for almost three years. But for me, at that time, I might still feel unhappy, but when I think about the adversity I've been through, why be unhappy? At the start, I really felt unhappy for a long time, it was like I fell into a deep abyss... But then actually, I can overcome these difficulties myself. I had two types of cancer. Kidney cancer was discovered first, stage three kidney cancer. Then, a few months later, I went to see a doctor, and the doctor referred me to the ENT department, and then I was diagnosed with nasopharyngeal cancer (laughs).	
2. Change in life values, and commitment on prioritizing one’s health		Case 16: I’ve endured (hard work) for nearly twenty years. The most important thing now is to take care of your health. Take care of yourself as much as possible. ... Your life is important. Once you have this illness, you will cherish yourself more. Before, you were willing to do anything, willing to do things without any hesitation, but now it feels like it's not worth it. If you really can't do certain things, then it's better to retire, take it easy... Eat better, don't just work endlessly and do more exercise... I used to give it my all, but now, I won't anymore...Otherwise, you'll just hurt yourself. The doctor told you not to exert too much force… I experienced it myself, when I exerted too much force, I had a nosebleed. Case 16: Because my weight dropped sharply (during treatment). I lost about twelve or thirteen kilos, you know. Now I've gained it back and I look bigger (less frail) now, walking feels a lot easier. When colleagues see me, they say, "Wow, you don't look like an old man anymore, you’re walking so fast."...When people tell me, I'm like, "What? Did I look like an old man?" Because I can't see those things myself. And also, I’m taking part in exercise, swimming. I have physical strength now, it turns out I can swim several laps, just like before, I've regained almost half of my strength back, so I have more confidence now. Case 14: I'm personally very optimistic... when I had cancer, at the beginning, I was a little unhappy, but it didn't affect me much. In the end, I think, everyone will eventually leave (laughs). Those that need to go will go. If I can have more time here, then I will live for a longer period of time.	
3. Self-distraction of cancer-related worries to cope with sleep disturbances		Case 20: Mindfulness really helped me... I follow along (to the video) and listen to it while relaxing, and I listen to relaxing music, like the sound of flowing water or ocean waves, or listen to the kind of music that helps you sleep. Then I sleep really well... It helped me get rid of my thoughts and relax, and I slept really well. So, I found out that mindfulness is really helpful for me, it helps me get rid of stress and the stress that I'm not aware of, it helps me focus on my body's reactions and state, and it helps me avoid thinking about unnecessary things. Case 30: Sometimes, before you go to bed, when you have nothing to do, you may look at the ceiling and start thinking... What will I do with my life in the future? ... That's why sometimes I listen to the radio before going to bed. I try to listen to it while sleeping... If I try to avoid things related to cancer and death, then I'll be better off.	
4. Realistic expectations towards sleep quality		Case 2: If I can't sleep, then I just get up. That's how it is, there's nothing you can do about it. Now, I just go with the flow, sleep as much as I can, right? If you keep thinking that you can't sleep, then it's even worse, right? So now, I live in the present moment. Well, it is what it is, just go with the flow. That's how I interpret it. But, of course, I still hope to find a way to sleep better. Case 20: I don't think it's that strict (to sleep 7-8 hours). I also think that's a time reference. Of course, if you sleep for seven to eight hours, that's ideal, it's a benchmark, a reference point. If you do not have enough energy after sleeping, or if you still feel pain after sleeping, then, that matters more. You can't just count how many hours you've slept.	
5. Pre-existing comorbid health conditions lead to sleep disturbance		Case 4: My respiratory system has never been good, I've had these problems since I was a child... since I was a baby. Because I mainly have to take the medication for my airways, prescribed by the doctor. And I also need to use an inhaler. I have to use it during the day and night. Case 2: Sometimes I feel that when I lie on my side, that specific area of the hip bone hurts, and it's so painful that I easily wake up. Also, because I’ve always had a herniated disc in my lower back, so there are issues with my lumbar bone as well. Previously, I didn't feel much pain in that area, but now I'm thinking maybe it's because my muscles have weakened (after cancer treatment), causing more pain in that area (when sleeping). Case 3: Actually, I don't think it has much to do with cancer, because even before I had cancer, I couldn't sleep well. It has been like this for many years. I think it has been a decade or more.

#### Patients with borderline or clinical psychological distress (Table 4.A)

Patients who had higher levels of psychological distress more often reported rumination about their progress towards recovery, and experienced greater concern about how negative emotions and poor sleep might impact their recovery. Consequently, they felt a heightened pressure to maintain good sleep quality. Patients also reported ongoing life stressors and lack of social support as triggers for sleep disturbances.

#### Rumination about recovery and returning to normal (4.A.1)

Patients with non-clinical and borderline or clinical psychological distress both experienced cancer-related side effects that negatively affected their sleep, however the latter group was found to fixate on their symptoms as an indication of poor recovery progress and inability to return to normal. A patient (Case 8) exhibited unbearable levels of discomfort from treatment side-effects, which prevented them from falling asleep easily. They perceived symptoms as uncontrollable and managing them to be an isolating experience, as they believed the symptoms would last indefinitely. While patients with non-clinical levels of distress tended to see treatment-related symptoms as a natural part of recovery, patients with higher distress levels seemed to find them overwhelming. One patient mentioned having once preferred death over facing their symptoms, particularly during the treatment period, which contributed to disturbing their sleep (Case 25). A patient (Case 15) was observed to exhibit upward comparison; as they had perceived their symptoms to be more severe when comparing with other patients at regular check-ups, which left them with feelings of uncertainty about their recovery.

#### Worry about negative emotions affecting cancer progression and subsequent thought suppression (4.A.2)

Patients with higher distress frequently expressed worry about whether having negative emotions arising from ruminating about cancer would further impact their health. For example, rumination of their side effects led to additional worry about how these negative emotions would affect their health condition (meta-worry) (Case 8). While conscious efforts to maintain a positive mindset by suppressing negative thoughts were made, patients also acknowledged that this was often challenging (Case 8). Another patient worried whether feelings of anger, triggered by arguments with their partner, would hinder the effectiveness of chemotherapy treatment. This worry in turn caused additional distress, which led them to avoid situations that lead to anger in order to better control their emotions (emotional suppression) (Case 1).

#### Worry about poor sleep affecting recovery, and subsequent pressure to achieve good sleep (4.A.3)

Many patients with higher distress exhibited concerns about the potential consequences of poor sleep, alike to the above reported effects of negative emotions on cancer recurrence or progression (Case 7). For example, a patient (Case 24) reported that maintaining good sleep was especially important given their history of cancer recurrence.

However, conscious efforts to fall asleep or by taking naps during the day to compensate for lost sleep was counterproductive and instead resulted in increased difficulty to sleep (Case 23). Anxiety over sleep concerns may also be reinforced by their sleep quality of the previous night, fearing their sleep difficulties would continue as they had already experienced it multiple nights in a row (Case 1). Patients also monitored bodily sensations, such as the level of comfort achieved in their sleeping environment (Case 27) or for any physical symptoms (Case 24), to determine the reasons behind their sleep difficulties.

#### High sleep reactivity to ongoing life stressors and lack of social support (4.A.4)

Apart from cancer-related factors, patients with higher distress were more likely to exhibit greater rumination towards other sources of stress such as family-related worries, planned engagements for the next day, and work stress, which contributed to increased difficulty in falling asleep (Case 26, 27). Likewise, it was commonly observed by patients that worry of being unable to sleep ultimately resulted in an increased failure to fall asleep (Case 32).

Alongside ongoing life stressors, patients with higher distress often reported having limited social support. For example, arguments that arose from a patient’s dissatisfaction towards the partner’s emotional and financial support towards the patient’s treatment expenses precipitated rumination on these issues before sleep (Case 1). Again, awareness of their negative emotions also became a source of worry for cancer progression. Alternatively, a patient (Case 31) cited the primary cause of her sleep disturbances, aside from cancer, was the burden and stress of being a full-time caregiver for a family member with intellectual disability.

#### Patients with non-clinical psychological distress (Table 4.B)

Patients who experienced lower levels of psychological distress typically demonstrated acceptance of their illness and treatment process, placed greater emphasis on moving forward with life after cancer, and expressed a shift in personal values to prioritize health. Simultaneously, they placed less fixation on achieving good sleep quality and were more likely to report comorbid health conditions diagnosed prior to cancer as contributors of sleep disturbance.

#### Acceptance, resilience and optimism towards cancer and treatment experience (4.B.1)

In contrast to those with higher distress, those with non-clinical distress generally attributed less emotional distress to their treatment side-effects. This was seen in Case 11, where an initial fear of potential radiotherapy side-effects evolved to a positive reappraisal of their cancer treatment journey after realizing that radiotherapy-induced damage was less than anticipated. Further, they were more likely to reframe their perspective by reflecting on the adversities they had faced in the past and used them as sources of motivation for increased personal growth to further overcome future difficulties (Case 14).

#### Change in life values, and commitment to prioritizing one’s health (4.B.2)

In contrast to those with higher distress, patients with less distress demonstrated a shift in values and priorities after cancer treatment, placing greater emphasis on caring for their health rather than fixating on their recovery progress. For example, a patient (Case 16) displayed a stronger focus on the positive aspects of their cancer treatment experience and demonstrated a commitment to actively seek ways to improve their health. They also expressed that it no longer feels worth it to exert themselves. Instead, they accepted their physical limitations after cancer, made necessary adjustments to their behavior accordingly, and embraced their new normal. Similarly, while one patient (Case 14) shared their perspective on the inevitability of death, they also indicated their commitment to making most of the present moment.

#### Self-distraction of cancer-related worries to cope with sleep disturbances (4.B.3)

While those with high distress engaged in avoidant strategies, such as disengaging from negative thoughts, those with non-clinical levels of distress conversely engaged in distraction of negative thoughts, by actively engaging in activities, such as mindfulness, or listening to the radio, that redeployed attention to external activities (Case 20; Case 30).

#### Fewer expectations towards sleep quality (4.B.4)

Compared with those with high distress, patients with non-clinical distress exhibited fewer expectations regarding their sleep quality. Instead, patients tended to accept sleep difficulties if they arose (Case 2). Some patients were not very worried about how much time they slept and whether it would affect their daily functioning (Case 20).

#### Pre-existing comorbid health conditions lead to sleep disturbance (4.B.5)

Many patients with non-clinical distress had pre-existing conditions that sometimes interfered with sleep, so were more accepting (Case 4). Cancer treatment was perceived by some to have worsened the symptoms of their comorbid conditions (e.g. herniated disc), further exacerbating their pre-existing sleep disturbance (Case 2). Consequently, many reported having become accustomed to sleeping poorly for many years (Case 3), suggesting that their sleep issues were unrelated to cancer.

### Common help-seeking behaviors in managing sleep disturbances (Table 5)

Irrespective of distress levels, both groups reported reluctance or opposition to seeking help from oncologists towards sleep disturbances. Patients either underestimated the severity of their sleep problems, believed it to have little effect on their daily lives or prioritized the treatment of other symptoms over sleep disturbance.

**Table 5:** Common help-seeking behaviors in managing sleep disturbances, narrative quotes

1. Lack of urgency to address sleep disturbances with oncologist	Case 2: Because I'm used to it. It’s been so long. I mean, before it was like that too, so now that you ask me (why I sleep poorly), I don't even remember why. I don't even think about why... Oh well, that's how it is, I gave up thinking about it, if I wake up then I will wake up... I wouldn’t say just because I sleep so little I don't have energy the next day, I don't feel like that…It's been a long time, these sleep problems have been going on for ten or twenty years. My situation (sleep problem) isn't too serious, I think. Case 32: I definitely value sleep quality, but if it (cancer) interferes with sleep quality, there should be something else involved that lead to this problem, so I would usually think about what the reason is and how it relates to sleep quality. So, sleep is a second priority for me. It's still important, but we have to find out the cause first. Case 9: I don't think it's particularly affecting me. Maybe there are other things to talk about (with the oncologist), for example maybe sometimes my hands and feet hurt for no reason, those things I might talk about, but not about sleep.
2. Preference for using Chinese medicine over Western sleep medication	Case 4: I don't want to take these bad things, because the more you take, the worse it is for your brain. I think sleeping pills, if you take them, your brain will possibly have a poorer memory. Case 33: I don't want to consider it (sleep medication). Natural therapy is better. All Western medication is toxic. And it can lead to addiction, I don't want that. Case 10: It's for treating this illness and nourishing the body. I'm doing acupuncture and taking Chinese medicine. So the doctor said that by regulating the body and improving the spleen and stomach, as well as improving Qi, my sleep quality will be a lot better. During the time of taking Chinese medicine, I woke up (in the middle of the night) much less often! Normally, I would go to the bathroom two or three times in between two hours, or once every two hours. But if I take the Chinese medicine, it changed to only once per night.
3. Perceived lack of support from oncologists for sleep and other health-related matters	Case 7: They think it's (poor sleep) normal. I think they believe that it's the same for everyone, they will have ups and downs (after treatment). They didn't mention much, but I don’t have much expectations from them. I just told them, "Doctor, I can't sleep, I wake up every two hours, and it's very difficult for me to fall asleep." ... but the doctor had no response. Case 28: The doctor didn't even let me see them, and they rushed me out in three minutes! ... The doctor didn't even let me say the word “worry"! They said, "If you're so worried, go find another doctor!" That's their response! Case 25: The side effects are so painful, I don't know why. It's been bothering me all this time and it hasn't gotten any better. It makes you initially feel very discouraged! Some doctors don't explain it clearly! ... Then they will say, "That's just how it is!" Like that. They will be with you for your whole life! They completely don't explain it, it's really terrifying!

#### Lack of urgency to address sleep disturbances with oncologist (5.1)

Patients with pre-existing sleep disturbance accepted poor sleep as a normal part of their lives (Case 2). They reported that they did not feel that sleep disturbance had a significant impact on their daily functioning, reflecting a lack of urgency in addressing sleep disturbances (Case 32). Some patients perceived other symptoms to be more important to address, such as unexplained pain in their hands and feet (Case 9).

#### Preference for using Chinese medicine over Western sleep medication (5.2)

Patients universally had negative opinions of sleep medication and Western medication in general, expressing concerns about its potential damaging effects on health and dependence. In contrast, there was widespread consensus in favor of seeking Chinese medicine as a method to treat sleep disturbances and other cancer-related symptoms (Case 4, Case 33)., they believed that traditional Chinese medicine (TCM) unlike allopathic medicine not only improves sleep quality, but also promotes overall health after undergoing cytotoxic cancer treatment, without having negative effects on the body (Case 10).

#### Perceived lack of support from oncologists for sleep and other health-related matters (5.3)

Reluctance to seek support was often influenced by previous experiences during regular check-ups with oncologists. For example, patients were informed that poor sleep was a normal post-treatment experience; and that there were few options available to manage sleep disturbances (Case 7). Some indicated disappointment or frustration with the oncologists’ responses, as they either felt that their concerns and worries were dismissed (Case 28), or that the oncologists had not provided enough information or guidance on symptom management post-treatment (Case 25). This often led to a sense of helplessness and uncertainty after their appointments.

## Discussion

This qualitative study compared the experiences and perceptions of sleep disturbances in cancer survivors between those without and with psychological distress. The findings revealed that irrespective of the presence of psychological distress, cancer survivors who have completed treatment continue to face persistent physical and psychological challenges, which contribute to sleep disturbances. Consistent with previous research, common triggers of sleep disturbances included treatment-related side effects [30, 48, 49], intrusive thoughts about cancer [50, 51], and fear of cancer recurrence or progression [52]. Non-cancer-related triggers of sleep disturbance were also identified, including a lack of routine, inactivity and poor sleep hygiene. As the majority of participants were unemployed, patients may have lower physical and cognitive engagement, which can lead to poorer sleep and irregular sleep–wake cycles [53] and disrupted sleep habits [29]. Notably, a number of patients had reported having long-standing sleeping difficulties that pre-dated their cancer diagnosis.

This qualitative study identified several notable differences between patients with various distress levels in how they adopted different coping responses towards common triggers of sleep disturbances, as well as differences in perceptions of sleep disturbances, which compounded existing sleep difficulties. Patients with borderline or clinical psychological distress more often mentioned rumination and worry about recovery after cancer, particularly regarding treatment side-effects, which often contributed to difficulty in falling asleep. High levels of worry regarding both negative emotions and poor sleep impairing cancer progression were often mentioned. This sleep-related worry prompted anxiety around feared negative consequences of sleep loss, such as cancer recurrence. Coping methods, including thought and emotional suppression, increased pressure to sleep, and heightened monitoring of sleep cues (e.g., bodily sensations for signs consistent with falling asleep) which are highly counterproductive, perpetuated sleep disturbances instead. Anticipation of sleep difficulties increases arousal around sleep which in turn delays sleep onset and confirms that the anticipation was justified. This then becomes self-sustaining. These findings align with Harvey’s cognitive model of insomnia [28], which proposes that the combination of sleep-related worry, selective attention and monitoring, and maladaptive safety behaviors (attempts to avoid or control unwanted thoughts) throughout the day contributes to increased arousal and interferes with sleep at night. However, worry about negative emotions arising when ruminating on cancer related side effects (meta-worry) or poor sleep potentially contributing to cancer progression or recurrence is also consistent with the newer metacognitive model of insomnia [54]. This model proposes that attentional bias towards sleep-related cues that interfere with sleep (primary arousal) may amplify the existing negative emotional valence (secondary arousal) associated with these thoughts, further exacerbating the inability to fall asleep. Such negative emotions feedback to further increase worry and intensify attentional bias. In the context of cancer, patients with high distress may be more vulnerable to sleep disturbances than the general population, as selective attention towards sleep-related worries or physical symptoms (e.g. pain) may amplify personally relevant fears, including cancer recurrence, progression and ability to return to normalcy.

Patients with borderline or clinical distress also mentioned nocturnal rumination about ongoing life stressors unrelated to cancer. These observations resemble trait sleep reactivity, which refers to an increased likelihood of developing insomnia in response to stressful events [55]. Trait sleep reactivity was not only found to moderate the effects of stress-induced intrusive thoughts on insomnia, but insomnia had also mediated the effect of trait sleep reactivity on depression [56]. Hence, it can be inferred that high levels of sleep reactivity may further exacerbate sleep disturbances by amplifying the impact of stress-induced or cancer-related intrusive thoughts in patients with higher distress levels; which in turn may also exacerbate existing depressive symptoms. Additionally, social support for this group was limited, which may further contribute to a sense of isolation, reduced emotional well-being and increased use of maladaptive coping strategies to manage sleep disturbances [57].

In contrast, patients with non-clinical distress exhibited noticeably less emotional distress in response to treatment-related physical symptoms. Their adoption of better adaptive mechanisms to manage the impact of cancer included a changed commitment to prioritizing one’s health, acceptance of their cancer treatment experience, while focusing on self-improvement rather than just recovery progress. This is concurrent with previous research that has shown that coping skills such as resilience, self-efficacy and emotion regulation may protect against psychological distress [58, 59] after treatment completion in cancer survivors. Observed capacities to adapt their actions to personal goals and values while accepting the reality of their adversities closely mirrors psychological flexibility, a core construct of the Acceptance and Commitment Therapy Model (ACT) [60]. This aligns with previous research that employed latent profile analysis which demonstrated that those in the low psychological flexibility class reported the highest levels of psychological distress, whereas those in the high psychological flexibility class reported the lowest levels of psychological distress [61]. Based on the ACT model, it can be inferred that patients with non-clinical distress have higher psychological flexibility; thus, they are able to experience and observe unpleasant, negative physical and psychological concerns about cancer and their inability to sleep, without having the urge to control them [62].

Additionally, while the borderline to clinical distress group engaged in thought suppression, the non-clinical distress group engaged in self-distraction and external attentional focus to cope with sleep disturbances. While it can be argued that both groups engaged in avoidant coping, some research has suggested that self-distraction is a predictor of better sleep quality [63], as diversion of attention is achieved by replacing an unwanted thought with another, which is thought to be a temporary reprieve to prevent the individual from re-engaging in worries and concerns [28]. This coping mechanism potentially enabled them to effectively reduce the impact of cancer-related worries on sleep disturbances. Differences in perceptions of sleep were also observed; patients with non-clinical distress had less concern over their sleep disturbances and had lower expectations of sleep quality. This may be accounted for by physical symptoms associated with pre-existing comorbid health conditions that had already contributed to sleep disturbances before cancer diagnosis, which were more commonly observed in the group with non-clinical distress. This is consistent with previous longitudinal research indicating that higher comorbidity is an explanatory variable for sleep disturbances in lung cancer patients [64]. As a result, although cancer-related factors may still trigger sleep disturbances for this group, the associated metacognitions surrounding cancer-related concerns and sleep-related worry may be less likely to be a contributor of poor sleep.

This qualitative study also captured help-seeking behaviors in response to sleep disturbances. Most patients, regardless of psychological distress levels, were reluctant to seek help from oncologists. This reluctance was influenced by an unfavorable view sleep medication specifically and Western medication in general, coupled with past negative experiences with oncologists when addressing their health-related concerns. This sample of Chinese patients preferred to use traditional Chinese medicine (TCM) to reduce side effects, aid recovery and enhance immunity after treatment; and deemed Western medication to be “toxic”. However, recent evidence has raised concerns regarding the safety of drug-herb interactions, particularly when combined with cancer treatment [65]. Additionally, reported negative interactions with medical staff are consistent with the service gaps identified in the overall survivorship care in Hong Kong, particularly in regards to unmet healthcare information needs [66, 67], which may be likely to be driven by low satisfaction or poor patient education [68]. Sleep disturbances are often presumed by both clinicians and cancer patients to be a transient response to cancer diagnosis and treatment, which ultimately self-resolves. Consequently, patients rarely discuss sleep disturbances with their oncology team [36]. Routine screening for sleep disturbances and more information regarding the benefits and limitations of different treatment options at oncology clinics should be provided to help patients make informed treatment decisions.

The findings have provided insights into the suitability of future interventions for patients with sleep disturbances. As sleep disturbances and psychological distress may not always present as a cluster, particularly during long-term survivorship, interventions that target only one area are likely to fail. A stepped-care program targeting patients with different distress levels may potentially offer a tailored approach to manage sleep disturbances. Cancer survivors, including those with low distress levels may benefit from physical activity programs that help to establish structure and increase activity in their daily routine [69], symptom management interventions to reduce discomfort-related causes of sleep disturbances [70], and encouragement to adopt greater externalizing focus and engagement in cognitively-taxing activities. Meanwhile, those with high distress are likely to benefit from interventions that target maladaptive emotion-focused coping strategies, such as sleep-related worry and metacognitions of worry, particularly those who are socially isolated. These may include acceptance and commitment therapy [54, 71], or cognitive behavioral therapy for insomnia [72], which has been recently found to produce similar effects on reducing depression compared with cognitive behavioral therapy for depression [73].

Our study has some methodological limitations. Sleep disturbance was only assessed once at recruitment, which may have unintentionally captured patients who had acute sleep disturbances affected by ongoing life stress. Future studies may implement repeated measurements over three months or longer in order to recruit patients with persistent sleep disturbances or chronic insomnia [74]. Furthermore, there was an unequal representation of patients with borderline to clinical or non-clinical levels of distress, which may affect the generalizability of the findings. However, the proportion of borderline to clinical levels of distress identified in the current study (39.4%) was similar to previous prevalence reports of head and neck cancer patients which constituted the majority of our sample. 21.1% and 48.9% of head and neck cancer patients with persistent poor sleep (PSQI > 5) were previously reported to have depression or anxiety (HADS > 7), respectively [75]. Thus, the unequal representation of patient groups in this study may be attributed to the overall lower prevalence of patients with depression or anxiety. Lastly, while the qualitative study captured various sleep disturbance experiences across different cancer types, majority of the sample consisted of breast, head and neck and gynecological cancers. Consequently, the unique experiences of sleep disturbances and treatment-related factors of other cancer types may not be adequately represented as clinical characteristics were not adjusted for. Future qualitative research may consider focusing on specific cancer types, cancer stages or treatment types to better understand how distress may uniquely impact sleep disturbances in different contexts.

In summary, while treatment-related side effects, intrusive thoughts about cancer, recurrence concerns and poor sleep hygiene may trigger sleep disturbances, patients with higher distress may be particularly vulnerable to maladaptive coping responses, such as increased worry around recovery after cancer, sleep-related worry, meta-worry, thought suppression, and high sleep reactivity, which may further perpetuate sleep disturbances. In contrast, those with non-clinical distress were more likely to experience persistent sleep disturbance as a result of coexisting physical symptoms related to other comorbid health conditions. Coupled with their reluctance in seeking professional help for supportive care services after cancer treatment, patients may not be driven to manage ongoing sleep disturbances. Future longitudinal studies may consider testing the effect of potential risk factors identified in contributing to sleep disturbances, as well as mediating pathways that operate across different distress levels. Pilot intervention studies are warranted in order to compare the effectiveness of customized approaches to manage sleep disturbances based on distress levels.

## Supplementary Information

Supplementary Material 1.

## Data Availability

The datasets generated during and/or analyzed during the current study are not publicly available due to privacy considerations but are available from the corresponding author on reasonable request.
