# Supplementary material for: Comparing the experiences of cancer survivors living with sleep disturbances between differing levels of psychological distress: a qualitative study
Source: BMC Psychiatry. 2024 Dec 2;24:869. doi: 10.1186/s12888-024-06344-4 (PMC11610178; doi:10.1186/s12888-024-06344-4)
Supplement: Supplementary file 1 — Supplementary Material 1. [file 12888_2024_6344_MOESM1_ESM.docx]

Appendix A. Questions asked in semi-structured interview

|  |  |
| --- | --- |
| 1)    Can you tell me your experience of having sleep problems? Could you please describe what it is like to have sleep problems, and what type of sleep problems you have? | |
|  |  |
| 2)    For how long have you had sleep problems? When did you start having them? If you have had sleep problems before your cancer diagnosis, were those different to your sleep problems after your diagnosis? | |
|  |  |
| 3)    What do you think contributed to your sleep problems? Can you think back to when you started to have sleep problems, and if anything can come to your mind that you believe triggered your sleep problems or worsened your sleep? | |
|  |  |
| 4)    How do sleep problems affect your daily life, or yourself in the long-term? For example, how does it affect you physically, behaviourally, emotionally, and socially? | |
|  |  |
| 5)    Have you ever noticed having particular thoughts and feelings when you are having sleep problems? What sort of thoughts do you think about, and how do you feel during the moment? Do you think those thoughts and feelings affect your sleep? | |
|  |  |
| 6)    What do you do when you have sleep problems? Have any methods helped to reduce sleeping problems, and how did they help you? | |
|  |  |
| 7)    Have you ever spoken to a medical professional about your sleeping problems? If so, did they recommend any medication? | |
|  |  |
| 8)    What does a “good night’s sleep” mean to you? Have you ever heard of sleep hygiene? | |
|  |  |
| 9)    Do you have any other thoughts you would like to share with me about your experience with having sleep problems? | |
